# Structure-function analysis of the retinoblastoma tumor suppressor protein – is the whole a sum of its parts?

**DOI:** 10.1186/1747-1028-2-26

**Published:** 2007-09-13

**Authors:** Frederick A Dick

**Affiliations:** 1 London Regional Cancer Program, London, Ontario, Canada; 2 Children's Health Research Institute, London, Ontario, Canada; 3 Department of Biochemistry, Schulich School of Medicine and Dentistry, London, Ontario, Canada

## Abstract

Biochemical analysis of the retinoblastoma protein's function has received considerable attention since it was cloned just over 20 years ago. During this time pRB has emerged as a key regulator of the cell division cycle and its ability to block proliferation is disrupted in the vast majority of human cancers. Much has been learned about the regulation of E2F transcription factors by pRB in the cell cycle. However, many questions remain unresolved and researchers continue to explore this multifunctional protein. In particular, understanding how its biochemical functions contribute to its role as a tumor suppressor remains to be determined. Since pRB has been shown to function as an adaptor molecule that links different proteins together, or to particular promoters, analyzing pRB by disrupting individual protein interactions holds tremendous promise in unraveling the intricacies of its function. Recently, crystal structures have reported how pRB interacts with some of its molecular partners. This information has created the possibility of rationally separating pRB functions by studying mutants that disrupt individual binding sites. This review will focus on literature that investigates pRB by isolating functions based on binding sites within the pocket domain. This article will also discuss the prospects for using this approach to further explore the unknown functions of pRB.

## Background

The retinoblastoma susceptibility gene (*RB-1*) was the first tumor suppressor gene to be cloned [1]. Since that time its encoded protein (pRB) has emerged as a key regulator of cell cycle entry and appears to be one of the most frequent targets for inactivation in human cancer [2-5]. The retinoblastoma protein is most frequently inactivated in cancer by the negative regulatory activity of cyclin dependent kinases [6]. Only in small cell lung carcinoma [7] and retinoblastoma [8] is the *RB-1 *gene a frequent target for direct mutation. Loss of heterozygosity at the *RB-1 *locus has been reported in many different sporadic cancers, suggesting that it is directly mutated outside of the lung and retina, but on a less frequent basis [9]. Based on pRB's prominent and ubiquitous role in cancer many investigators have focused their efforts on trying to determine its biochemical function. A fundamental component to this type of investigation is the evaluation of mutant alleles to determine which aspects of cell physiology require pRB. Ideally the analysis of very specific mutants will determine which protein interactions account for how pRB functions. This review will focus on the efforts that have been made to rationally separate different aspects of pRB's function in proliferative control and cancer.

**Figure 1:**
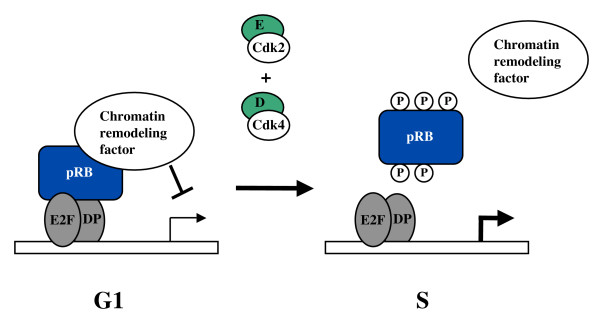
**Transcriptional control of the cell cycle by pRB**. In G1 pRB is bound to E2Fs and masks their ability to activate transcription. In turn, pRB can recruit a number of chromatin remodeling factors that can further inhibit the initiation of transcription. Mitogen signaling then activates cyclin/Cdk complexes that phosphorylate pRB in late G1 and early S-phase leading to the disassembly of this complex and the activation of transcription of genes needed for progression through S-phase.

Given that the focus of this review is on the dissection of pRB function, some reports will inevitably be omitted because they lack a structural component. By no means is this is meant to diminish their validity, it is hoped that all aspects of pRB function will eventually fit into a framework of defined protein interactions, unfortunately not all are at this stage. In general terms, pRB has an established role in mediating a G1 arrest in development and in response to many growth regulatory signals [5]. Some examples are DNA damage [10], or growth inhibiting cytokines such as TGF-β [11]. In addition, pRB plays a key role in the permanent cell cycle exit of differentiating cells and this has been demonstrated both in cell culture and *in vivo *using gene targeted mice [12,13]. The Retinoblastoma protein plays an essential function in permanent cell cycle arrest and differentiation of adipocytes [14], myotubes [15], osteoblasts [16], and neurons [17]. In mechanistic terms, much of pRB's ability to control the cell cycle has been linked to its ability to regulate transcription [4,18]. The RB protein binds to E2F transcription factors and blocks their ability to induce transcription of genes that are needed to advance the cell cycle. In turn, once pRB is brought to a promoter by an E2F it can recruit chromatin remodeling proteins such as histone methyltransferases [19], DNA methyltransferases [20], histone deacetylases [21,22], and helicases [23] among others to further repress transcription [24]. Stimulation of cell cycle entry results in phosphorylation of pRB by cyclin/Cdk complexes and causes the release of E2Fs and chromatin regulators at the G1 to S-phase transition (Figure 1).

Beyond the simple model elaborated in Figure 1 where pRB represses transcription in G1 to control S-phase entry, it should be noted that recent work on pRB to validate this mechanism is not without its controversies. However, one common theme to pRB function is that it works as an adaptor protein that can nucleate complexes containing multiple interacting partners. For this reason, understanding the consequence of the loss of one type of pRB interaction in isolation holds enormous promise to characterizing how pRB works in controlling proliferation, or other functions, that make it a tumor suppressor.

## Nature's bounty; using tumor-derived mutations in *RB-1 *to guide functional analysis

**Figure 2:**
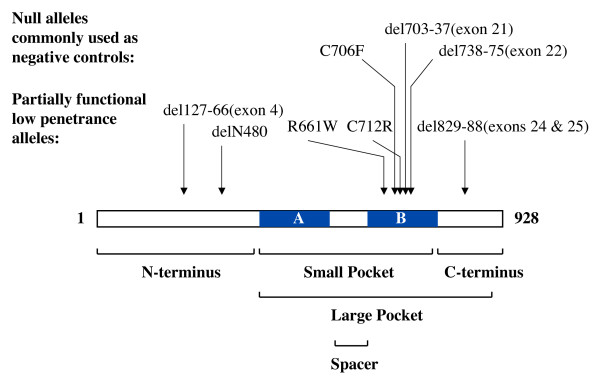
**Commonly used RB-1 mutations and pRB domain structure**. The open reading frame of pRB is shown along with the locations of the A and B parts of the pocket. Arrows indicate the positions and alterations found in commonly studied low penetrance and high penetrance mutant alleles. Below the open reading frame, structural regions of pRB are identified along with their approximate locations.

Before describing what has been learned from studying pRB through a structure-function approach it is worth understanding what mutant resources are available. For example, oncogenic mutations in Ras are clustered at codons 12 and 61 and these mutants have revealed the importance of the GTP bound state [25]. This insight was made possible by the fact that oncogenic mutations discretely cripple intrinsic GTP hydrolysis while leaving interactions with downstream effectors intact. Generation of mutations such as the S17N substitution has created a dominant negative form of Ras whose over expression blocks endogenous Ras signaling [26]. At this point there are no known cancer causing mutations in *RB-1 *that offer discrete separation of activities. Much of the effort to dissect pRB function using cancer causing alleles has come from mutations found in retinoblastoma families with partially-penetrant inheritance [27-30], particularly the R661W substitution (Figure 2). However, these alleles encode proteins that contain multiple interaction defects. Furthermore, there has been relatively limited use of gain-of-function and dominant negative alleles of *RB-1 *to help clarify its function despite efforts to develop these reagents [31-35]. Instead, a number of tumor derived *RB-1 *mutants have actually emerged as excellent negative controls!

In addition to Ras, another relevant comparison of mutations is with p53. The *TP53 *gene is the most frequently mutated gene in cancer [36]. Cataloguing hundreds of tumor derived missense changes has been invaluable, as it has revealed the importance of the DNA binding domain in p53 tumor suppressor function. Some of these alleles have also turned out to have dominant negative activity. At present the vast majority of mutations found in retinoblastoma patients are deletions or nonsense changes, suggesting that these are null alleles [8,37]. There are 35 known cancer causing missense mutations in *RB-1 *from retinoblastoma patients and most of these alleles have only been reported once [37]. Taken together, this suggests that the paucity of partially defective *RB-1 *mutants has prevented the type of analysis of pRB that has played a crucial role in understanding other oncogenes and tumor suppressors. In short, nature has not been very generous in providing mutations to aid in analyzing the mechanism of pRB function.

## Cut and paste, elucidating the domain structure of pRB

The retinoblastoma protein does not possess any commonly recognized DNA binding or protein interaction domains, although crystallographic data demonstrates that pRB contains two cyclin-folds and it has similarity to other proteins with this structure [38,39]. Our present knowledge of the domain structure of pRB has been determined by deletion mapping. These efforts have defined the large and small 'pocket' domains (Figure 2). The 'small pocket' was initially identified as the minimal fragment of pRB that can interact with viral proteins like SV40 TAg, HPV E7, and adenovirus E1A [40-42]. Crystal structure data indicates that the A and B halves of the small pocket each represent a single cyclin fold and they interact to form a dumbbell shaped globular domain [38]. Experiments designed to define the minimal growth-suppressing domain identified a slightly bigger piece of pRB that is called the 'large pocket' [43]. The large pocket is also capable of complementing pRB's tumor suppressor activity *in vivo *when expressed in place of the full-length protein [44]. In addition, the cancer-causing missense mutations in *RB-1 *that have been described are found mostly within the large pocket and this has focused efforts most intensively on this domain. Puzzlingly, there are two other proteins in the RB-family called p107 and p130 that have extensive similarities to pRB, particularly within the small pocket [45,46]. Despite this similarity, there is little experimental evidence to suggest these proteins have tumor suppressive functions on their own as *p107*^-/-^*;p130*^+/- ^and *p107*^+/-^*;p130*^-/- ^mice are not tumor prone [45]. This has further focused the question on what does the pRB pocket domain do that p107 and p130 can't do?

Dissection of interactions and separating functions within the large pocket domain has been one of the rate limiting steps to advancing our understanding of pRB by a structure-function approach. Initially, efforts to mutagenize pRB were guided by sequence conservation and these have lead to a number of insightful reports that separate different aspects of pRB function [47-49]. However, crystal structures of the pRB small pocket have revealed that many of the most conserved amino acids are buried and this suggests why some of these mutants have partial loss of function for multiple interaction types [38]. Generation of synthetic mutant alleles of *RB-1 *that seek to create discrete defects have been greatly aided by the crystal structure of the small pocket. This has provided a detailed description of which amino acids are on the surface of this domain. Furthermore, recent co-crystal structures containing pRB and pieces of E2Fs have further indicated the sites of important protein-protein interactions within the large pocket region [50-52]. The use of this information to generate mutations and guide experimentation into the mechanism of pRB function will hopefully pave the way for a more rational understanding of how pRB controls cell proliferation and suppresses cancer development.

The perspective that is presented above is meant to emphasize that our mechanistic understanding of pRB is still in its infancy because we are only now developing the tools to discover how pRB works, or to definitively test many of the long-standing models of its function. To further emphasize the importance of mapping and disrupting individual protein-protein interaction sites in mechanistic studies of pRB, the ensuing sections of this review will be organized around individual protein-protein interaction sites or activities mediated by the large pocket domain. To truly focus this review on structure-function analysis of pRB, the work that is discussed will highlight reports that attempt to dissect function within the large pocket domain, and not merely demonstrate its involvement. In this way the goal is not only to discuss recent work, but also to present it from a viewpoint that will reveal important gaps in our mechanistic knowledge of pRB by attempting to describe its function as a sum of a few discrete parts.

## Regulation of E2F transcription factors, is it the key to pRB function or just another in a long list?

E2F1 was one of the first pRB interacting proteins to be identified [53,54]. It is now known that pRB interacts with the first four members of the E2F family [55]. Each of these E2Fs needs to partner with a DP subunit to stably interact with pRB and to bind to E2F promoter elements [4]. Each of E2Fs 1, 2, and 3 are potent stimulators of cell cycle progression from G1 to S-phase and are referred to as 'activator' E2Fs [18]. Direct interaction with pRB blocks this activating function because pRB masks its activation domain [56-60]. Over expression of activator E2Fs can bypass pRB's growth suppressing activity [61]. Likewise, ectopic proliferation in Rb deficient mouse embryos can be suppressed by reducing E2F activity by crossing to E2F1, 2, or 3 knock out strains [62-64]. For these reasons this interaction is viewed as central to how pRB functions in proliferative control and as a tumor suppressor gene. From a structure-function perspective, investigating the properties of pRB mutants that are deficient for E2F binding has tested the importance of this interaction. The data that has emerged from these studies will be discussed below and it shows that E2F regulation is necessary for a maximal cell cycle arrest by pRB, but it also indicates that pRB has other growth regulating functions.

Initial reports of the disruption of E2F regulation by pRB used the low penetrance pRB allele, R661W. This mutant protein is defective for binding to E2F transcription factors in *in vitro *binding assays [28,47], and endogenous interactions have been evaluated in knock-in mice where this protein shows a dramatic reduction in binding to all activator E2Fs. In cell culture assays, this mutant retains some activity in proliferative control even though transcriptional regulation of E2Fs has been lost [47,65]. In addition, Saos-2 cells that are derived from an osteosarcoma are able to undergo aspects of bone differentiation and senescence in response to R661W [47]. The functional effects of the R661W mutation in a knock in mouse model are similar [66]. These animals develop to a slightly later stage of gestation than the knock out, despite showing complete deregulation of E2F transcription and control of fibroblast proliferation. The authors offer a number of carefully examined paradigms for differentiation in erythrocyte and macrophage lineages that show R661W retains at least partial activity. Thus, the R661W mutation may have more defects than loss of E2F regulation, but it still retains some of pRB's growth regulating activity.

Two reports describe the structure of the small pocket bound to the pRB binding domain of an E2F [50,52]. These have provided molecular insight into this important interaction. Substitution of amino acids that mediate E2F and pRB interactions has revealed that clean removal of this E2F binding site can eliminate transcriptional repression of E2F reporters [67,68]. Similar to what has been found with R661W, discrete loss of E2F binding to pRB does not abrogate all proliferative control activity as most models of pRB function (such as that pictured in Figure 1) have predicted [67,68].

**Figure 3:**
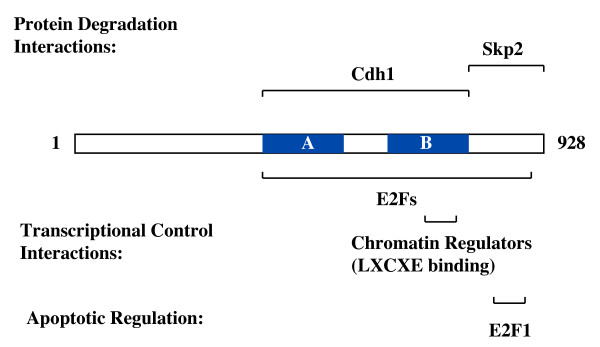
**Binding sites for E2Fs and other proliferative control activities**. The open reading frame of pRB is shown with locations of the A and B parts of the pocket. Regions of contact that have been mapped for E2F transcription factors, chromatin regulators, and protein degradation machinery that are involved in controlling proliferation are diagramed.

If removing E2F binding does not eliminate G1-S regulation by pRB, but only cripples it, what functions remain to control the cell cycle? One candidate that has been proposed to mediate pRB arrest that is distinct from E2F regulation is p27. Using mutants of pRB that have impaired E2F binding, p27 levels were shown to be elevated during senescence induction in Saos-2 cells [49]. More recently, an insightful report by Ji et al. showed that cells induced to express wild type pRB are capable of arresting in G1 before repression of E2F targets takes place [69]. These authors further demonstrate that the immediate activity in cell cycle arrest comes from the pRB C-terminus' ability to interfere with Skp2 targeting p27 for degradation. The R661W mutant retains this function, reconciling this activity with previous observations of cell cycle regulation by this mutant. These experiments establish a direct link between pRB and the control of cyclin/Cdk activity that is separate from transcriptional regulation of cyclin and Cdk genes. A more detailed evaluation of the mechanism of Skp2 regulation by pRB reveals that APC^Cdh1 ^and Skp2 simultaneously bind to pRB [70]. This interaction targets Skp2 for degradation. Analysis of the interaction sites for these molecules with pRB reveals that Skp2 contacts the C-terminal region of pRB [69] and Cdh1 makes use of the LXCXE binding cleft in combination with E2F contacts in the large pocket [70]. Based on the similarity of contact sites used by APC and Skp2 in degradation, and those by E2F/pRB/chromatin regulating complexes, it appears that pRB has two discrete cell cycle control mechanisms that are mediated by similar regions of the large pocket (Figure 3). This implies that in G1 two separate populations of pRB are engaged in distinct growth regulating mechanisms.

In the process of removing E2F binding from pRB by mutagenesis it was also determined that pRB retains the ability to bind to E2F1 using a separate interaction site [67,68]. This E2F1 only, or 'Specific' binding site, is located exclusively in the C-terminal portion of pRB [67]. Furthermore, apoptotic induction by E2F1 can be inhibited by a pRB mutant that is deficient for binding to all other E2Fs [67]. Mutation of the E2F1 'Specific' binding site inhibits this activity even when the other E2F binding site is present [71]. Chau et al. have shown that a pRB mutant that is incapable of regulating cell cycle advancement and is only capable of binding to E2F1, can inhibit apoptosis induced by DNA damage [68]. This suggests that E2F1 regulation may have a role in DNA damage signaling independent of cell cycle regulation by pRB. Recently, the C-terminus of pRB was co-crystallized with fragments of E2F1 and DP1 [51]. This structure reveals the molecular contacts between a well-conserved portion of pRB that is not found in p107 or p130 and a region of E2F1 that is different from the other E2F family members. In addition, swap experiments exchanging the contact site on E2F1 with the analogous region of E2F3 demonstrate that this interaction is truly unique to E2F1 [71]. Mechanistically, it remains to be determined how pRB uses this interaction with E2F1, however, the identification of a novel pRB-E2F interaction through structure-function analysis reveals that there are new regulatory pathways that have yet to be explored.

Much emphasis has been placed on the central role of pRB-E2F interactions in cell cycle control. Remarkably, experiments that investigate the requirement for these interactions reveal that they are important, but alone can not explain how pRB controls proliferation. Future work will need to quantitatively define the contribution of these non-E2F mechanisms of cell cycle control. The R661W mutation suggests that non-E2F and E2F dependent cell cycle control mechanisms can be separated, however the pleiotropic nature of this mutation leaves us unsure how much each contributes. Alleles that discretely remove each arrest mechanism alone, or both together, will help answer how much each contributes to pRB's overall function. More importantly, do E2F and Cdh1/Skp2 interactions account for all activity in cell cycle control by pRB or are their even more mechanisms? It will also be necessary to use model organisms to investigate how much E2F regulation contributes to pRB's role as a tumor suppressor compared with Cdh1/Skp2 or others. This type of analysis will ultimately determine how central E2F regulation really is to pRB's function or whether our models of pRB-E2F regulation in cancer need to be reconsidered.

## From viral transformation to the histone code, the LXCXE binding cleft has something for everyone

**Figure 4:**
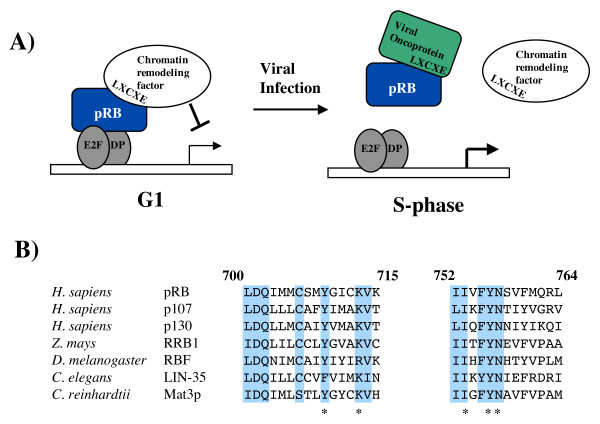
**pRB and the LXCXE motif**. (A) A repressor complex composed of an E2F transcription factor, pRB, and a chromatin regulator is shown in G1. The interaction between pRB and the chromatin regulator is mediated by the peptide sequence LXCXE on the chromatin remodeling factor and the LXCXE binding cleft on pRB. Viral proteins like TAg, E7, and E1A can disrupt this complex because they contain an LXCXE motif. Disruption of this pRB-containing complex can advance the cell cycle into S-phase. (B) The LXCXE binding cleft on pRB is a shallow groove formed between two parallel helices. The amino acid sequence of these helices in human pRB is aligned to the analogous sequences from pRB-family proteins from humans, corn, fruit flies, worms, and algae. Shaded amino acids are identical between all protein comparisons. The asterisk denotes amino acids whose side chains make direct contact with LXCXE in the crystal structure reported by Lee et al. [38].

As described above, the small pocket region of pRB was initially mapped as the minimal domain capable of binding to Adenovirus E1A, SV40 TAg, and HPV E7 [40]. Evaluation of the transforming properties of these viral oncogenes revealed that a peptide motif called LXCXE was necessary for pRB binding and transformation [72-77]. The convergent evolution of these very different viruses highlights the importance of RB family inactivation in viral transformation. Molecular insight into this interaction was revealed when the LXCXE motif was co-crystallized with the small pocket of pRB [38,78]. Beyond revealing the intermolecular contacts between the LXCXE motif and the pRB small pocket, this work also revealed that the contact site of the LXCXE motif on pRB is one of the most well conserved structural features of pRB family proteins [38] (Figure 4). This region of the pocket is often called the LXCXE binding cleft. The conservation of amino acids in the LXCXE binding cleft implies that protein interactions mediated by it are a key component of pRB's function.

Amino acid sequence conservation provides a straightforward rationale for studying the LXCXE binding cleft on pRB. Unfortunately, understanding how pRB uses LXCXE type interactions in proliferative control remains ill defined. One reason is that there are 28 proteins that have been reported to interact with pRB using an LXCXE motif (Table 1), raising the question of whether all of these interactions are necessary for proliferative control events mediated by pRB, or whether some are needed for more specialized functions. It is beyond the scope of this review to discuss how each of these molecules is proposed to partner with pRB, however, most cellular proteins that possess an LXCXE motif are believed to work with pRB in transcriptional regulation. Some of these proteins have an enzymatic activity or other property that can modify chromatin structure, these include BRG1, Brm, DNMT1, HDAC1 and 2, HP1, RBP2, RIZ1, and Suv39h1 [19-23,79-83]. Others have no clear biochemical activities, but contribute to repression of transcription in cell culture assays, or are themselves known transcription factors. These include CtIP, EID1, ELF1, HBP1, RBP1, and UBF [82-90]. It is difficult to generalize the other LXCXE motif proteins, but they participate in functions as varied as DNA replication and apoptosis while many have no clearly defined functions. Examination of Brm, RBP1, RBP2, and HBP1 irrespective of pRB has revealed roles for them in regulating proliferation or maintaining terminal differentiation [91-94].

**Table 1:** Proteins that use an LXCXE interaction motif to bind to pRB

**Protein**	**Biochemical Functions**	**References**
AhR	Aryl hydrocarbon receptor	[158]
ASK1	Ser/Thr kinase	[159]
Bog	Unknown	[160]
BRG1	ATP dependent helicase	[161]
Brm	ATP dependent helicase	[110]
CtIP	Transcriptional repressor	[84]
Cyclin D1	kinase regulatory subunit	[162]
Cyclin D2	kinase regulatory subunit	[163]
Cyclin D3	kinase regulatory subunit	[164]
DNA Pol delta	DNA polymerase	[165]
DNMT1	DNA methyltransferase	[20]
EID-1	Unknown	[85] [86]
ELF1	Transcription factor	[87]
HBP1	Transcriptional repressor	[88] [89]
HP1	trimethyl histone H3 binding protein	[19]
HDAC1	histone demethylase	[21] [22] [80]
HDAC2	histone deacetylase	[81]
Hec1	Unknown	[166]
Hsp75	Heat-shock protein	[167]
p202	Unknown	[168]
p204	DNA replication	[169]
RFC p145	DNA replication	[170]
RBP1	Chromatin remodeling/transcriptional repression	[82]
RBP2	histone demethylase	[82]
RIM	Unknown	[171]
RIZ	methyltransferase	[83] [172]
Suv39h1	methyltransferase	[19]
UBF	PolI transcription factor	[90]

Given the broad possibilities for how pRB can use LXCXE type interactions (because of the vast number of interacting proteins that contain this motif), recent efforts have focused on mutating the LXCXE binding site on pRB and examining the defects caused by loss of all LXCXE interactions with pRB. Surprisingly, the ability to control cell proliferation remained largely intact in Saos-2 cell cycle arrest assays [95-98]. However, in some assays of permanent cell cycle arrest, mutations in this region of pRB were shown to allow cell cycle re-entry [96,98]. Recently, the generation of a knock-in mutant mouse strain has been described in which the LXCXE binding site on pRB has been disrupted [99]. These mice are viable, implying that cell cycle exit and differentiation occurs normally during development. It remains to be seen if there are proliferative control defects in these mice or if they are cancer prone because of them. However, the defect that was highlighted in this report was genomic instability. Disruption of pRB-LXCXE interactions caused defects in heterochromatin condensation due to diminished histone H4-K20 trimethylation. Chromosomal regions normally characterized by this modification become fused causing errors in mitosis. Thus, one *in vivo *manifestation of defective LXCXE interactions is abnormal chromatin structure.

Another approach to identify proteins that cooperate with pRB by contacting the LXCXE binding site has been to purify pRB containing protein complexes. Isolation of the fruit fly RBF proteins with a complex of other transcriptional regulators has offered insight into the assembly of a transcriptional repressor module called dREAM [100,101]. Sequence comparisons have revealed that this is an evolutionarily conserved complex whose components are found in distantly related organisms including humans, flies, and *C. elegans*. This complex of proteins contains subunits that are dependent on the LXCXE binding cleft for interaction with pRB in *in vitro *binding experiments [100]. The use of ChIP assays to identify target promoters and RNAi to deplete individual members of this complex has revealed that it functions to block transcription of a vast number of developmentally regulated genes and is also involved in controlling DNA synthesis in *Drosophila *oocytes [100-103]. Mutations in the LXCXE binding cleft in knock-in mice would be expected to disrupt assembly of this complex, so how can dREAM be essential for regulating so many transcriptional targets while the knock-in mice remain viable? A recent clue has come from the isolation of endogenous protein complexes containing the p130 protein [104]. This work has revealed that endogenous human dREAM components only assemble with p130 and not other pocket proteins, suggesting that human pRB differs from pocket proteins found in lower eukaryotes because it does not participate in the dREAM complex [104].

In summary, mice with an LXCXE binding cleft mutation have defective chromatin structure and this is in agreement with chromatin regulating enzymes using an LXCXE motif to bind to pRB [99]. However, the lack of obvious cell cycle phenotypes caused by disruption of LXCXE interactions in cell culture and knock-in mice reveals that there is not a universal requirement for them in all aspects of pRB's proliferative control activity [95-99]. The investigation of transcriptional repression mechanisms using model organisms like *C. elegans *and *Drosophila *has provided tremendous insight into the RB family of proteins. However, their RB gene families are slightly different in that they do not possess an exact orthologue of human pRB. Because of these differences, the dREAM complex seems unlikely to be a key cellular partner of pRB in mammals [104], leaving in question how pRB uses the LXCXE binding cleft? The fact that mice with LXCXE cleft mutations are viable creates a perplexing contradiction since its maintenance creates susceptibility to the pathogenic effects of DNA tumor viruses [105]. Since the LXCXE binding cleft on pRB is one of the most well conserved features of RB family proteins [38] it must have an important function. Determining the cellular context where LXCXE interactions with pRB are essential is the first step to understanding this aspect of pRB function. Further exploration of phenotypes in LXCXE binding cleft mutant mice should guide this analysis. Identifying the exact LXCXE dependent partners that cooperate with pRB remains a daunting task. A true understanding of how pRB uses LXCXE interactions will likely require some rationalization of the many possible binding partners.

## Induction of differentiation and transcriptional activation; is pRB a switch hitter?

In the preceding sections little effort has been made to discriminate between cell cycle arrest and terminal differentiation. Both involve a block in cell proliferation; however, terminal differentiation also includes a significant change in gene expression patterns as these cells take on a specialized function. In addition to participating in cell cycle exit, pRB has been proposed to augment expression of cell type specific genes during differentiation [12,13]. In this way pRB can aid in transcriptional repression as well as activation, depending on the cellular context. This section of the review will focus on the mechanisms that pRB participates in to facilitate transcription in differentiation.

The activity of transcription factors that induce differentiation can be augmented by pRB. These include MyoD, CBFA1/RUNX2, C/EBPs, NF-IL6, Pax transcription factors, and nuclear hormone receptors [14,16,106-112]. In addition, pRB dependent inhibition of proteins like EID-1, Id2, and RBP2 has also been shown to activate transcription during differentiation [85,86,93,113]. So how does pRB induce differentiation? From a structure-function perspective the best evidence for pRB's role in differentiation has come from a synthetic mutant pRB called Δ663 that substitutes five amino acids in the pRB pocket starting at codon 663 to the peptide sequence NAIRS. This protein is defective for E2F binding, transcriptional repression, and induction of a G1 arrest. However, expression of this mutant induces markers of both muscle and bone differentiation [47]. It has also been reported that mutations in the LXCXE cleft of pRB prevent it from fully activating MyoD dependent transcription during myogenesis [114]. Because of the structural component underlying the data on pRB's role in muscle and bone differentiation, these paradigms will be discussed further.

In the case of muscle, pRB is necessary for MyoD to maximally induce late markers of differentiation [15,106]. Precisely how pRB augments MyoD is not known but it seems to involve influencing phosphorylation of MEF2C that is necessary for maximal transcription of late markers [115]. In the case of bone differentiation, direct interaction between pRB and HES1 with RUNX2/CBFA1 induces DNA binding and enhanced transcriptional activation of bone specific transcriptional targets [116]. Conversely, induction of muscle or bone differentiation can be aided by pRB's ability to antagonize HDAC1, EID-1, and RBP2 in transcriptional regulation. HDAC1 is able to inhibit MyoD in cycling cells and upon induction of differentiation pRB titrates HDAC1 away from MyoD allowing it to activate transcription. Similarly, EID-1 can inhibit p300/CBP acetylase activity that is necessary for MyoD to activate transcription. At the onset of differentiation, pRB binds to EID-1 and targets it for degradation thereby allowing MyoD to recruit p300 to stimulate transcription of muscle specific genes [85,86]. In a similar manner, pRB has been proposed to interact with RBP2 and remove it from promoters where it inhibits transcriptional activation through its H3-K4 demethylase activity [93,117]. In assays of CBFA1/RUNX2 dependent activation, pRB's stimulatory effect is abrogated by siRNA depletion of RBP2 [93]. Likewise, MyoD's inability to stimulate differentiation in pRB deficient fibroblasts can be rescued by simultaneous depletion of RBP2 [93]. These data strongly suggests that RBP2, HDAC1, and EID-1 along with pRB play critical roles in differentiation through antagonism.

These mechanisms offer insight into how pRB can function in aspects of differentiation beyond a mere G1 arrest. However, many questions remain regarding pRB's role in differentiation. Since the work highlighted above does not reveal much common ground between these different mechanisms, but a single mutant (Δ663) is capable of inducing many of them, does pRB have one type of protein-protein interaction just for differentiation? Or are there many, subtle activation mechanisms where no one mechanism is essential? If the second possibility is true, then does this imply that pRB is really just a cell cycle regulator and its role in differentiation is indirect? When considering this possibility it is important to remember that CKI proteins p16 and p21 can block proliferation, but can not enhance transcription by MyoD [15]. Only pRB has this dual ability, suggesting that it must have additional activities in differentiation beyond G1 arrest.

In order to move forward and come to a firm conclusion about how pRB works in differentiation, new structure-function data is imperative to define which interaction surfaces are essential. The Δ663 mutant is defective for E2F binding as well as contact with LXCXE proteins suggesting that neither of these is likely to be necessary for transcriptional activation [47]. Curiously, each of HDAC1, EID-1, and RBP2 are proposed to contact pRB using an LXCXE motif [22,82,85,86]. This apparent contradiction raises the question of whether LXCXE interactions are really necessary in differentiation? Rb deficient embryos that are rescued by a normal placenta can develop to birth where they die due to defects in myogenesis [118], revealing a place in development where pRB plays an essential function in this differentiation paradigm. Isaac et al. report that homozygous mutation of the LXCXE binding cleft results in a viable mouse [99], indicating that LXCXE interactions with pRB are not be essential for muscle development. However, Benevolenskaya et al. have shown that RBP2 can interact with the R661W mutant of pRB and mutation of the LXCXE motif on RBP2 has only a minimal effect on the interaction with pRB [93]. This suggests that possession of an LXCXE motif does not mean that it is essential for mediating an interaction with pRB. In short, structure-function analysis of pRB's role in differentiation has wet our appetite about what it may do, but has left us unsure of how it actually participates.

Future work in this area would be greatly aided by a class of pRB mutants that can block proliferation, but not induce differentiation, rather than the reciprocal properties that have already been reported. This type of mutant will focus our attention on which contacts are essential for pRB dependent transcriptional activation.

## Regulation by post-translational modifications, just an on-off switch or are there different settings?

The retinoblastoma protein is a target for many post-translational modifications. The most extensively studied aspect of pRB regulation is phosphorylation [4], however, it can be acetylated [119] and sumoylated [120] as well. In addition, apoptotic signaling can modify pRB function by proteolytic cleavage [121].

Regulation of pRB by phosphorylation was first observed as a mobility shift in SDS-PAGE where pRB is faster migrating in G1 phase cells and slower migrating in S, G2, and M-phase [122-125]. This behavior gave rise to the terms hypophosphorylated (pRB) and hyperphosphorylated (ppRB) when referring to these bands in western blots. Binding assays to study pRB interactions with E2Fs, chromatin regulators, and other binding partners have revealed an almost uniform preference for binding to the hypophosphorylated form of pRB [4,24]. This indicates that pRB binds to its interacting partners in G1 when it is underphosphorylated implicating this as the active form. Phosphorylation at the beginning of S-phase then prevents pRB from interacting with other proteins until the end of mitosis when it is dephosphorylated [126]. Based on this data, phosphorylation offers a relatively clear on-off mechanism for pRB regulation and this has been discussed in great depth in many previous reviews [3-7,18]. However, a considerable number of experiments raise the question of how closely is this golden rule actually followed?

The earliest studies of pRB phosphorylation have used cell synchronization procedures to enrich for cells containing active pRB in G1 and inactive ppRB from the other phases [55,125]. The presence of pRB/E2F complexes in other phases of the cell cycle has often been described as a technical artifact of the synchronization procedure, or the lag between new synthesis of pRB and its phosphorylation. A number of recent reports have demonstrated by ChIP that pRB/E2F complexes can be detected on DNA in S-phase cells [127,128]. Furthermore, analysis of RBF/dE2F complexes from fruit flies indicates that some are resistant to inactivation by cyclin/Cdks and these repress transcription at all stages of the cell cycle as revealed by synchronous cell divisions in the developing eye disc [129,130]. Taken together this indicates there are circumstances where pRB family proteins are resistant to inactivation by cyclin/Cdks despite being exposed to high levels of their activity.

**Figure 5:**
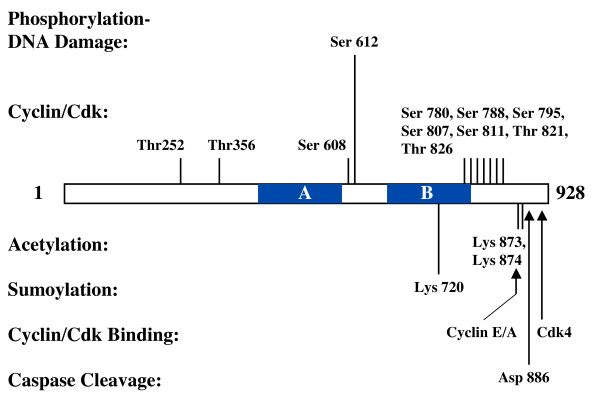
**Post-translational modifications of pRB**. The open reading frame of pRB is depicted with the A and B regions of the pocket indicated. Cyclin/Cdk phosphorylation sites that have been investigated by mutagenesis are shown as are sites of phosphorylation following DNA damage. The location of acetylated and sumoylated lysines are indicated. Arrows indicate the approximate location of Cdk4 and cyclin E binding sites that are needed for optimal phosphorylation of pRB. Caspase cleavage of pRB occurs on the C-terminal side of aspartate 886, removing amino acids 887 to 928.

Phospho-peptide maps have suggested as many as 21 phosphorylation sites on pRB [131,132]. Through sequence analysis, human pRB contains as many as 16 potential cyclin/Cdk phosphorylation sites containing the S/T-P consensus [31]. These phosphorylation sites are located in three general areas of the pRB open reading frame, the N-terminus near the beginning of the pocket domain, the spacer region, and the C-terminal domain just after the small pocket (Figure 5). In G1, cyclin D/Cdk4 complexes make use of a Cdk4 binding site in the extreme C-terminus of pRB that is required for efficient phosphorylation [133]. Ablation of all three D-cyclin genes reduces phosphorylation on residues 249, 252, 807, 811, and 826 of pRB suggesting that they are primarily the targets of D associated kinases [134]. Cyclin E/Cdk2 becomes active near the G1-S boundary and uses an interaction motif called RxL or Cy to recognize pRB and phosphorylate the remaining sites [135-137]. There are a number of potential RxL motifs in the C-terminus of pRB, but mutagenesis indicates that two adjacent motifs that begin with lysine residues 870 and 873 are the most important [135]. These experiments reveal a concerted mechanism in which D and E cyclin/Cdk complexes bind to pRB at discrete locations in the C-terminus and phosphorylate it at other locations. A number of reports have suggested that the C-terminus of pRB is largely disordered and flexible [38]. This allows substrate recognition through binding while flexibility ensures access to phospho-acceptor sites.

Using mutagenesis 11 of the possible 16 Cdk phosphorylation sites have been substituted to alanine to prevent inactivation of pRB [31-33]. Experiments using these constructs have revealed that this non-phosphorylatable form of pRB can block cell proliferation in fibroblasts and tumor derived cell lines [32,33,138]. Examination of pRB mutants that have only a subset of these substitutions reveals that there is no single phosphorylation site that plays a critical role in the inactivation of E2F binding to pRB [31,32,139]. However, interactions at the LXCXE binding cleft can be regulated by a subset of phosphorylation sites in the C-terminal region of pRB [140,141]. Curiously, arrest of Saos-2 cells with non-phosphorylatable pRB can be bypassed by cyclin/Cdks without phosphorylating pRB [138]. Thus, phosphorylation of pRB to control advancement of the cell cycle may be dispensable. In addition, expression of non-phosphorylatable pRB in mammary epithelia of transgenic mice initially causes slowed proliferation during development, but eventually ductal infiltration of the fat pad catches up and exhibits hyperplasia and in some cases carcinogenesis [142]. The authors of this study argue that non-phosphorylatable pRB would also remain active for the inhibition of E2F induced apoptosis and this could explain its oncogenic properties. Regardless of the interpretation, it is clear from these transgenic studies that phosphorylation of pRB is not ubiquitously required for cell division to occur.

In addition to post-translational modifications that occur in response to mitogenic signals, pRB is also modified in response to DNA damage. Acetylation of lysines 873 and 874, as well as phosphorylation of serine 612 occurs rapidly in response to double strand DNA breaks [143,144]. The immediate implication of these findings is that post-translational modifications on pRB can occur under circumstances other than proliferative control. In fact, DNA damage activates G1 and S-phase arrest mechanisms that dephosphorylate ppRB at cyclin/Cdk sites [128,145,146]. This implies that modifications added to pRB following DNA damage result in a gain in function. The exact mechanism of action that these modifications initiate is not clear. Phosphorylation of Ser612 has been reported to enhance binding to E2Fs as an anti-apoptotic mechanism [144] while acetylation of 873, 874 has been suggested to release E2Fs to induce cell death [143]. To further add to the confusion of how acetylation of pRB regulates its function, Nguyen and co-workers have shown that pRB becomes acetylated during muscle differentiation [147]. Using a non-acetylatable mutant where lysines 873 and 874 are changed to arginines, they demonstrated that this mutation leaves E2F transcriptional regulation intact. Interestingly, the resulting muscle cells are susceptible to re-entry into S-phase when stimulated by serum suggesting that acetylation is necessary for pRB to enforce permanent cell cycle exit. Clearly more work is needed to define the role of pRB 'activation' in differentiation or following DNA damage, but these studies illustrate that there is more to the regulation of pRB than a simple on-off mechanism at the G1-S boundary. One implication of acetylation of lysines 873 and 874 is that there may be a hierarchy of pRB regulation where acetylation blocks phosphorylation because the Cy motifs at this same position are no longer recognized by cyclin E/Cdk2. This could be advantageous in differentiation and DNA damage because it would prevent promiscuous phosphorylation of pRB and ensure cell cycle arrest.

The most recently discovered modification of pRB is sumoylation. Sumoylation is a ubiquitin like modification that has recently been implicated in numerous biological functions, including the induction of senescence [148]. The retinoblastoma protein has been reported to be sumoylated in the small pocket near the LXCXE binding cleft at lysine 720 [120]. The exact mechanistic consequence of modifying pRB with sumo is not fully understood, however, it has been proposed to compete with proteins that bind to the LXCXE cleft. In other studies of sumoylation, the overall effect of this modification on p53 and RBP1 is an induction of senescence [149,150]. Whether sumoylation of pRB plays a role in the induction of senescence is not known, but the pRB LXCXE binding cleft has been proposed to interact with chromatin regulators to establish repressive heterochromatin as part of a permanent arrest [151]. This raises the question of whether HP1 and other chromatin regulators can contact pRB if sumo is interfering with access to this surface.

Tumor necrosis factor alpha (TNF-α) stimulates inflammation and apoptosis [152]. It signals to activate so called 'initiator' caspases that cleave substrates as part of a signaling cascade that ultimately leads to the activation of 'effector' caspases that ultimately kill the cell. Caspase cleavage of pRB occurs at an intermediate step of TNF-α signaling and results in the irreversible removal of the C-terminal 42 amino acids [153,154]. This is defined as an intermediate step in cell death because expression of a caspase resistant mutant of pRB inhibits progression of signaling and the effector caspases are never activated [153,154]. Using a knock-in mouse model where the DEADG recognition sequence in pRB is changed to DEAAE (called pRB-MI), Chau et al. showed that intestinal epithelia are uniquely resistant to TNF-α mediated cell death [155]. Furthermore, crossing the pRB-MI mice to *p53*^-/- ^mice results in frequent colonic adenomas that are rare in *p53*^-/- ^mice. This work suggests that pRB is a critical target in apoptotic induction by TNF-α and disruption of this pathway in the intestine allows damaged cells to survive and begin to form tumors [156]. Unfortunately, the mechanistic details of how pRB is regulated by proteolysis are less well understood. Two possibilities are that cleavage eliminates binding to a pro-apoptotic factor that pRB normally sequesters and its release allows cell death can to ensue [154,155], alternatively removal of the last 42 amino acids destabilizes pRB so that it's expression is eventually lost [153]. In this way cells would effectively behave as if they were pRB deficient and E2F1 would be liberated to kill cells. Given that TNF-α can induce cell death without the need for new protein expression, the former possibility seems more likely although there remains much to be done to understand this mechanism [155].

To summarize our present knowledge of post-translational regulation of pRB, most attention has been focused on phosphorylation and this modification has been shown to block pRB's growth inhibitory properties. Despite the preponderance of evidence for negative regulation of pRB by phosphorylation, it is clear that exceptions exist to this regulatory mechanism. Perhaps other regulatory modifications can 'mark' pRB to retain function in the face of cyclin/Cdks as described above, or inactivate it without phosphorylation. The answer to how pRB regulation is impacted by multiple signaling pathways will need to await further investigation. Never the less, these different types of modifications are revealing further complexity to an already multi-functional protein.

## Conclusion

A common theme in this review is that a large number of proteins contact a relatively small region of pRB. Mutations that affect the pocket domain can simultaneously disrupt many aspects of pRB and have made dissecting interactions within this domain very difficult. Perhaps the reason why a vast majority of tumor-derived alleles of *RB-1 *eliminate the pocket domain completely [8] is because they represent the easiest way to disrupt everything that pRB does. Never the less, with a multi-functional protein like pRB where so much is mediated by just the large pocket domain, it is even more important to define how different interacting proteins can act together with it to control cell growth. Without this level of understanding we will never be able to say how pRB really works.

Despite the challenges of a structure-function based approach to pRB, discussing its function based on different protein interaction sites, or structural domains, as described above readily covers most aspects of pRB's known role in proliferative control and cancer. In nearly each case the use of mutants with defined defects reveals that the prevailing view of pRB function is part of how it works but unlikely to be the whole explanation. These structure-function approaches to study pRB have revealed many unexplained activities and highlight areas for further investigation. Just how important are these unexplored areas? A recent paper by Binne et al. shows that without Cdh1, pRB has almost no ability to induce a G1 cell cycle arrest [70]. Likewise, Sellers et al. have shown a dramatic separation of proliferative and differentiation activities [47]. Thus, a number of these emerging areas are likely quite central to pRB function but are simply less well understood.

Structure-function approaches offer a reliable means to examine how proteins interact with one another to carry out various cellular processes. Combined with genetic analysis that defines sufficiency and necessity, the purpose of individual protein-protein interactions can be very effectively defined. Despite using this approach, we still struggle to draw firm conclusions about what makes pRB a tumor suppressor. The properties of the low-penetrance allele R661W suggest that E2F interactions are an essential component of this function. However, since this allele is only partially penetrant, and some carriers of this mutation can live cancer free [157], is it telling us that E2F binding is only a small part of tumor suppression by pRB? Does this implicate Cdh1/Skp2 binding in tumor suppression because it is part of the proliferative control mechanism that functions in the absence of E2F control in the R661W mutant? Or should we exclude Cdh1/Skp2 interactions because they are not deficient in R661W? Clearly more data on tumor predisposition caused by partially defective pRB molecules is essential to draw firm conclusions about this crucial question. Since there are a limited number of tumor derived mutations to choose from, generating more synthetic alleles with discrete defects is likely the only way forward. For example, if gene-targeted mice carrying the LXCXE cleft mutation turn out to be tumor prone, then we would conclude that LXCXE interactions are part of pRB's tumor suppressor function. This would undoubtedly motivate more effort to identify precisely how pRB uses this type of interaction compared with another binding site that is excluded by a similar tumor incidence study.

This type of systematic approach to investigating pRB tumor suppression could be brought to bear on many pRB interactors and binding sites. However, as lists like that in Table 1 reveal, pRB interacting proteins are numerous [24]. A potential opportunity to better focus our efforts on what makes pRB a tumor suppressor may also come from comparisons with the related pocket proteins p107 and p130. As stated earlier, these molecules share extensive similarity with pRB in the pocket domain, yet on their own neither appears to meet the genetic definition of a tumor suppressor gene. Focusing on proteins that interact with pRB and not p107 and p130 could help to guide our selection of interactors that are most relevant in cancer. By this way of thinking, a complex like APC^cdh1 ^would hold considerable appeal because it selectively binds pRB over p107 and p130 even in *in vitro *binding assays [70]. Alternatively, the dREAM complex that we now know only contacts p130 *in vivo *[104] would be a less appealing candidate to cooperate with pRB in tumor suppression. Advancing our understanding of pRB function in this most crucial of questions will clearly require multiple approaches. The investigation of endogenous pRB protein complex composition, combined with structure-function analysis of discrete mutant alleles of the *RB-1 *gene, offers a logical means to separate pRB function into distinct parts to define its role in cancer.

## Competing interests

The author declares that they have no competing interests.
